# Vector flow mapping analysis of left ventricular energetic performance in healthy adult volunteers

**DOI:** 10.1186/s12872-016-0444-7

**Published:** 2017-01-09

**Authors:** Koichi Akiyama, Sachiko Maeda, Tasuku Matsuyama, Atsushi Kainuma, Maki Ishii, Yoshifumi Naito, Mao Kinoshita, Saeko Hamaoka, Hideya Kato, Yasufumi Nakajima, Naotoshi Nakamura, Keiichi Itatani, Teiji Sawa

**Affiliations:** 1 Department of Anesthesiology, Kyoto Prefectural University of Medicine, 465 Kajii-cho, Kamigyo, Kyoto 602-8566 Japan; 2 Emergency Medicine, Kamigyo, Japan; 3 Cardiovascular Surgery, Kyoto Prefectural University of Medicine, Kamigyo, Kyoto Japan; 4 Department of Anesthesiology, Kansai Medical University, Hirakata, Japan; 5 Department of Statistical Genetics, Kyoto University, Kamigyo, Kyoto Japan

**Keywords:** Vector flow mapping, Energy loss, Kinetic energy, Energetic performance index, Vortex

## Abstract

**Background:**

Vector flow mapping, a novel flow visualization echocardiographic technology, is increasing in popularity. Energy loss reference values for children have been established using vector flow mapping, but those for adults have not yet been provided. We aimed to establish reference values in healthy adults for energy loss, kinetic energy in the left ventricular outflow tract, and the energetic performance index (defined as the ratio of kinetic energy to energy loss over one cardiac cycle).

**Methods:**

Transthoracic echocardiography was performed in fifty healthy volunteers, and the stored images were analyzed to calculate energy loss, kinetic energy, and energetic performance index and obtain ranges of reference values for these.

**Results:**

Mean energy loss over one cardiac cycle ranged from 10.1 to 59.1 mW/m (mean ± SD, 27.53 ± 13.46 mW/m), with a reference range of 10.32 ~ 58.63 mW/m. Mean systolic energy loss ranged from 8.5 to 80.1 (23.52 ± 14.53) mW/m, with a reference range of 8.86 ~ 77.30 mW/m. Mean diastolic energy loss ranged from 7.9 to 86 (30.41 ± 16.93) mW/m, with a reference range of 8.31 ~ 80.36 mW/m. Mean kinetic energy in the left ventricular outflow tract over one cardiac cycle ranged from 200 to 851.6 (449.74 ± 177.51) mW/m with a reference range of 203.16 ~ 833.15 mW/m. The energetic performance index ranged from 5.3 to 37.6 (18.48 ± 7.74), with a reference range of 5.80 ~ 36.67.

**Conclusions:**

Energy loss, kinetic energy, and energetic performance index reference values were defined using vector flow mapping. These reference values enable the assessment of various cardiac conditions in any clinical situation.

## Background

Blood flow in a healthy human left ventricle forms an energetically efficient vortex [1, 2]. This vortex facilitates inflow into the ventricle, minimizes the dissipation of energy, preserves momentum, and redirects the flow toward the left ventricular outflow [3–7]. Valvular heart disease and ischemic heart disease change the vortex configuration and increase dissipative energy loss (EL) [8, 9]. The intraventricular vortex and its energetic efficiency affect the patient’s outcome [10, 11]. For example, diastolic EL increases in aortic regurgitation proportionally to its severity [9], and the energy loss index provides independent and prognostic information additional to that derived from conventional measures of aortic stenosis severity [10]. In addition, in pediatric cases, EL is associated with an impaired relaxation of the ventricle among patients with Fontan circulation [12].

Several intracardiac flow visualization technologies have emerged recently, among which is vector flow mapping (VFM), a novel echocardiographic technology [13–15]. VFM enables visualization of the intraventricular flow velocity vector using color Doppler and speckle tracking data and to estimate the flow dissipative EL [16, 17]. Hayashi et al. have provided EL reference values for children [18], but those for adults have not yet been established. The aim of this study was to use VFM technology to establish a reference value for left ventricular flow dissipative EL in healthy adult volunteers to allow the assessment of a patient’s cardiac condition during the perioperative period using transthoracic echocardiography (TTE). Further aims were to establish reference values for kinetic energy (KE) and a new index, the energetic performance index (EPI), of the left ventricle in healthy adults.

## Methods

### Study population

The study was approved by the institutional review board (ERB-C-437) of Kyoto Prefectural University of Medicine, Kyoto, Japan, and written informed consent was obtained from all participants. The participants were 42 and 12 healthy men and women, respectively, aged 20 to 44 years, with no particular medical history or chronic disease and a structurally normal heart and sinus rhythm, who volunteered to participate in the study between November 2015 and June 2016. Exclusion criteria were poor quality echocardiographic images, and heart valvular disease or wall motion abnormality on echocardiographic examination.

### Transthoracic echocardiography

TTE was performed using an ultrasound machine, Prosound F75 Premier (Hitachi, Tokyo, Japan) with a 2.5 MHz sector probe, in accordance with the recommended method of the American Society of Echocardiography [19]. Two-dimensional cineloop images of the apical long-axis view were stored with the VFM configuration. The Nyquist limit for two-dimensional color Doppler imaging was increased to minimize the aliasing phenomenon; at the same time, the region of interest was kept large enough to include the left ventricular myocardium so that we could trace the ventricular wall to calculate intraventricular flow vector in VFM.

### Data collection

Clinical data including sex, age, height, body weight, and body surface area (BSA) were collected. The echocardiographic measurements collected included heart rate (HR), left ventricular end diastolic diameter, left ventricular end systolic diameter, left ventricular fractional shortening (LVFS), peak blood flow velocity at the mitral valve during early diastole (E) and late diastole (A), the E/A ratio, and peak myocardial velocity at the septal mitral annulus during early diastole (e’) and late diastole (a’) and the E/e’ ratio.

### Offline analysis by vector flow mapping

The stored ultrasound images from the Prosound F75 Premier were transferred to a computer for analysis with VFM software. The one cardiac cycle image for analysis was selected using two consecutive QRS complexes of the electrocardiogram as the beginning and end points. Whenever the aliasing phenomenon was recognized, the aliased areas were manually corrected with phase shift. The left ventricular endocardial boundary was manually traced to detect left ventricular wall motion by speckle tracking. Based on the VFM images, intracardiac EL values and the KE in the left ventricular outflow tract (LVOT) were calculated. There are three types of intracardiac EL: mean EL over one cardiac cycle (ELcycle), mean systolic EL (ELsys), and mean diastolic EL (ELdia). All three types of EL were calculated. Intracardiac EL values and the mean KE over one cardiac cycle in the LVOT (KEcycle) were averaged over three cardiac cycles.

### Principles of vector flow mapping and flow energy loss

VFM enables the evaluation of intracardiac flow and calculation of intracardiac EL and KE. This technology uses both color Doppler images and speckle tracking images. The velocity vectors of each pixel can be calculated using a continuity equation from the left and right side boundaries. The calculated velocity vectors are integrated according to a weight function [16, 17].

Intracardiac EL can be calculated from the following equation [17]:

$$ \boldsymbol{Energy}\ \boldsymbol{Loss}={\displaystyle \int}\mu \left\{2{\left(\frac{\partial u}{\partial x}\right)}^2+2{\left(\frac{\partial v}{\partial y}\right)}^2+{\left(\frac{\partial u}{\partial y}+\frac{\partial v}{\partial x}\right)}^2\right\}dA, $$

where μ is the viscosity of the blood, *u* and *v* are velocity components along the Cartesian axes (*x* and *y*), and A is the area of the unit of the grid.

As the equation indicates, EL is the total of squared differences between neighboring velocity vectors. It increases at points where the size and direction of velocity vectors change. For example, EL is likely to increase due to turbulent flow, such as at a site after aortic stenosis. The EL values are expressed in W/m because VFM images are two-dimensional.

The KE in the LVOT can be calculated from to the following equation:

$$ \boldsymbol{K}\boldsymbol{E}={\displaystyle \int}\frac{1}{2}\rho {v}^2\times vdL, $$

where ρ is the density of the blood (1060 kg/m^3^), *v* is the velocity vector of the blood flow, and dL is an increment of the cross-sectional line.

In addition, we have devised a new index, the EPI, which is useful for assessing the cardiac condition, effectiveness of treatment, and outcome of surgery. EPI is defined as follows:

$$ \boldsymbol{E}\boldsymbol{P}\boldsymbol{I}=\frac{KEcycle}{ELcycle} $$

### Reproducibility

Intra-observer variation in ELcycle and KEcycle were assessed in 10 randomly selected subjects, with the second analysis performed 2 weeks after the first analysis. Inter-observer variation was assessed by comparing the two quantitative measurements made by the first echocardiographer with those of the blinded second echocardiographer. Reproducibility was assessed by Bland–Altman analysis.

### Statistical analyses

Continuous variables are expressed as mean ± standard deviation. Reference ranges for each energetic parameter were calculated from the 2.5% and 97.5% values. Correlations between ELcycle, ELsys, ELdia, and KEcycle and age, height, weight, BSA, HR, and echocardiographic measurements were evaluated using Pearson’s correlation coefficients. Stepwise multivariate linear regression analyses were performed to analyze the independent variables that correlated with EL and KE. The analyzed variables were age, height, weight, BSA, HR, LVFS, E, A, and e’. Interaction between the independent variables was analyzed by stepwise multivariate linear regression analysis, setting the level of significance to *p* < 0.2. Regression equations for ELcycle, ELsys, ELdia, and KEcycle were analyzed based on the multivariate linear regression analysis. The statistical analyses were performed using JMP software version 12.0.1 (SAS, Cary, NC, USA). The significance level was set at *p* < 0.05.

## Results

### Demographic and echocardiographic data

TTE was performed in 54 volunteers. However, four women were excluded due to the poor quality of their echocardiographic images, leaving 50 volunteers to be included in this study. Their demographic and echocardiographic data are shown in Table 1.

**Table 1:** Demographic and Echocardiographic Data of 50 volunteers

Male	42 (84%)
Age (years)	29.5 ± 4.8
Height (cm)	170.8 ± 7.3
Weight (kg)	65.5 ± 10.9
BSA (m^2^)	1.76 ± 0.16
HR (bpm)	65.3 ± 9.6
LVEDD (mm)	41.9 ± 5.3
LVESD (mm)	26.9 ± 4.3
LVFS	0.36 ± 0.07
E (cm/s)	66 ± 17
A (cm/s)	38 ± 8
E/A	1.8 ± 0.5
e’ (cm/s)	11.9 ± 1.8
a’ (cm/s)	7.7 ± 1.8
E/e’	5.6 ± 1.4

Data are shown as means ± Standard Deviation

*BSA* body surface area, *HR* heart rate, *LVEDD* left ventricular end diastolic diameter, *LVESD* left ventricular end systolic diameter, *LVFS* left ventricular fractional shortening

### Vortex pattern analyzed by VFM

**Fig. 1:**
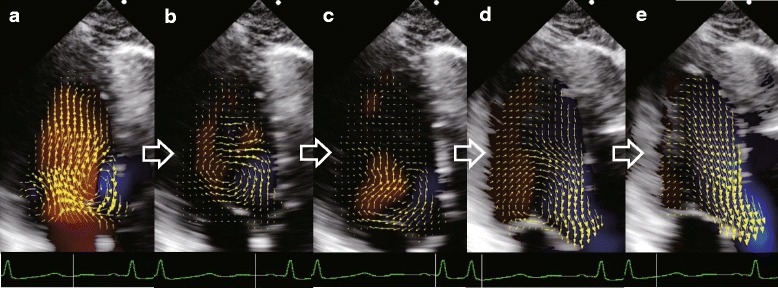
Example of a vector flow mapping image of a healthy volunteer. **a** The strong clockwise rotating vortex and the weak counterclockwise rotating vortex are shown during early diastole. **b** The weak counterclockwise rotating vortex diminishes, and the strong clockwise rotating vortex is in the mid-cavity of the left ventricle during mid-diastole. **c** The clockwise rotating vortex is now in the base cavity of the left ventricle due to the atrial contraction during late diastole. **d** Vortex momentum facilitates the ejection flow during early systole. **e** All of the flow from the whole left ventricular cavity is directed to the left ventricular outflow tract

A normal vortex pattern as shown in Fig. 1 (Case #1) was observed in all 50 volunteers. A clockwise rotating vortex formed during diastole and the ejection flow streamed out smoothly to the aorta (Fig. 1).

### Mean energy loss over one cardiac cycle

**Fig. 2:**
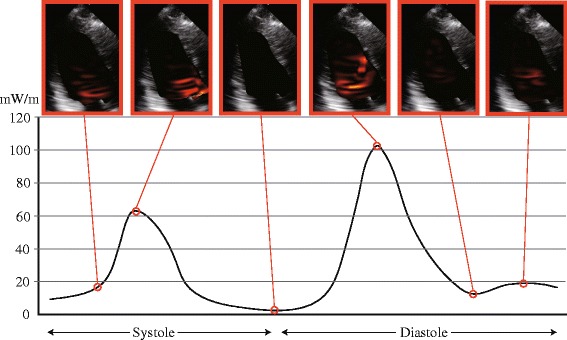
Example of energy loss images and a graph of a healthy volunteer. The energy loss images are superimposed on apical long-axis echocardiography views. Brightness indicates energy loss. The time phases are, from the left, early systole, mid-systole, isovolumetric relaxation phase, early diastole, mid-diastole, and late diastole. The systolic peak of the graph is due to the flow acceleration from the left ventricular cavity into the left ventricular outflow tract. The velocity vectors are aligned toward the outflow tract, demonstrated on the energy loss image by the bright area around the outflow tract. The diastolic peak of the graph is due to the dissipative transmitral inflow. The inflow forms vortices that minimize the energy loss

**Fig. 3:**
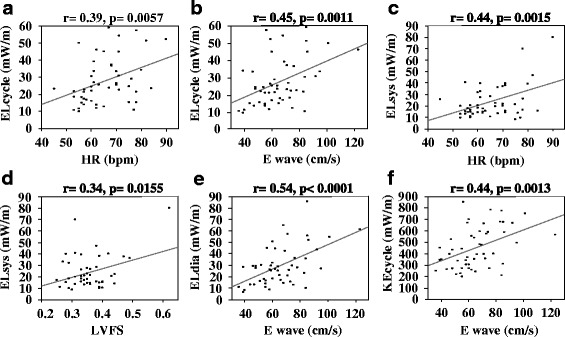
Correlations between energetic performance and other parameters. **a** Average energy loss over one cycle (ELcycle) and heart rate. **b** Average energy loss over one cycle (ELcycle) and E wave velocity **c** Mean systolic energy loss (ELsys) and heart rate (HR). **d** Mean systolic energy loss (ELsys) and left ventricular functional shortening (LVFS) **e** Mean diastolic energy loss (ELdia) and E wave velocity. **f** Average kinetic energy over one cycle (KEcycle) and E wave velocity

A typical graph of EL over a cardiac cycle is shown in Fig. 2 (Case #1). ELcycle, the mean value of EL over one cardiac cycle, is simple to use for assessing cardiac condition. The range of ELcycle was from 10.1 to 59.1 mW/m, with a mean of 27.53 ± 13.46 mW/m (male 27.44 ± 13.83, female 27.99 ± 12.14; Table 2). No significant difference was observed between male and female mean values. The reference range for ELcycle was 10.32 ~ 58.63 mW/m. there were statistically significant correlations between ELcycle and E (*r* = 0.448, *p* = 0.001) and HR (*r* = 0.385, *p* = 0.006) (Table 3 and Fig. 3). According to the multivariate analysis, the independent predictors of ELcycle were E (*p* < 0.0001) and HR (*p* < 0.0001), with the following regression equation:

**Table 2:** Analyzed Energetic Parameters of 50 volunteers total and gender-separated

Energetic Parameters	total	male	female
ELcycle (mW/m)	27.53 ± 13.46	27.44 ± 13.83	27.99 ± 12.14
ELsys (mW/m)	23.52 ± 14.53	23.88 ± 14.94	21.68 ± 12.82
ELdia (mW/m)	30.41 ± 16.93	29.93 ± 17.57	32.96 ± 13.75
KEcycle (mW/m)	449.74 ± 177.51	432.81 ± 165.85	538.60 ± 220.71
EPI	18.48 ± 7.74	18.98 ± 8.23	15.86 ± 3.66

Data are shown as means ± Standard Deviation

*ELcycle* mean energy loss over a cardiac cycle, *ELsys* mean value of systolic phase energy loss, *ELdia* mean value of diastolic energy loss, *KEcycle* mean kinetic energy over a cardiac cycle in the left ventricular outflow tract, *EPI* energetic performance index

**Table 3:** Correlation between the energetic parameters and other variables

Variables	ELcycle		ELsys		ELdia		KEcycle	
	*r*	*p*	*r*	*p*	*r*	*p*	*r*	*p*
Age	−0.970	0.503	0.114	0.430	−0.196	0.172	−0.035	0.809
Height	−0.135	0.351	−0.056	0.697	−0.161	0.265	−0.195	0.175
Weight	−0.116	0.422	−0.037	0.797	−0.147	0.309	−0.084	0.563
BSA	−0.133	0.358	−0.05	0.730	−0.163	0.257	−0.132	0.360
HR	0.385	0.006	0.438	0.002	0.264	0.064	0.049	0.738
LVEDD	0.085	0.557	−0.025	0.861	0.139	0.334	0.006	0.969
LVESD	−0.022	0.881	−0.209	0.145	0.111	0.442	−0.042	0.771
LVFS	0.159	0.269	0.341	0.016	−0.004	0.978	0.068	0.639
E	0.448	0.001	0.076	0.598	0.542	<.0001	0.442	0.001
A	0.227	0.112	0.139	0.334	0.207	0.150	0.083	0.567
e’	0.118	0.416	−0.016	0.915	0.166	0.250	0.266	0.062

*ELcycle* mean energy loss over a cardiac cycle, *ELsys* mean value of systolic phase energy loss, *ELdia* mean value of diastolic energy loss, *KEcycle* mean kinetic energy over a cardiac cycle in the left ventricular outflow tract, *BSA* body surface area, *HR* heart rate, *LVEDD* left ventricular end diastolic diameter, *LVESD* left ventricular end systolic diameter, *LVFS* left ventricular fractional shortening, *r* correlation coefficient, *p* value

$$ ELcycle=-46.720+0.430\times E+0.706\times HR $$

(adjusted *R* ^2^ 0.4211, *p* < 0.0001).

The coefficient estimates and standard errors for each independent variable, and the intercept and residual standard error for each energetic parameter, are shown in Table 4.

**Table 4:** Multivariate regression equation for predicting each energetic parameters

Variables	ELcycle			ELsys		
	CE	SE	*p*	CE	SE	*p*
Intercept	−46.720	12.69701	0.0006	−41.858	17.0963	0.0182
HR	0.706	0.155377	<0.0001	0.597	0.212634	0.0073
E wave	0.403	0.08589	<0.0001	0.155	0.108463	0.1594
LVFS				45.328	30.81608	0.1481
Age						
	RSE 10.23956			RSE 12.92186		
	A*R* ^2^ 0.4211			A*R* ^2^ 0.20866		
	*p* < 0.0001			*p* < 0.0032		
Variables	ELdia			KEcycle		
	CE	SE	*p*	CE	SE	*p*
Intercept	−39.733	18.98064	0.0417	155.307	89.26415	0.0883
HR	0.753	0.195887	0.0004			
E wave	0.583	0.108115	<0.0001	4.495	1.317788	0.0013
LVFS						
Age	0.582	0.395543	0.1479			
	RSE 12.70637			RSE 160.9024		
	A*R* ^2^ 0.4365			A*R* ^2^ 0.1783		
	*p* < 0.0001			*p* 0.0013		

*ELcycle* mean energy loss over a cardiac cycle, *ELsys* mean value of systolic phase energy loss, *ELdia* mean value of diastolic energy loss, *KEcycle* mean kinetic energy over a cardiac cycle in the left ventricular outflow tract, *HR* heart rate, *LVFS* left ventricular fractional shortening, *CE* coefficient, *SE* standard error, *RSE* residual standard error, *AR* ^*2*^ adjusted *R* ^2^

### Mean systolic energy loss

ELsys, the mean value of systolic phase EL, is a useful parameter for assessing the pathological cardiac condition at the systolic phase, such as systolic anterior motion, mitral regurgitation, and aortic stenosis. The range of ELsys was from 8.5 to 80.1 mW/m (male 23.88 ± 14.94, female 21.68 ± 12.82; mean 23.52 ± 14.53 mW/m; Table 2). No significant difference was observed between male and female mean values. The reference range for ELsys was 8.86 ~ 77.30 mW/m. ELsys showed statistically significant correlations with HR (*r* = 0.438, *p* = 0.002) and LVFS (*r* = 0.341, *p* = 0.016) (Table 3 and Fig. 3). According to the multivariate analysis, the independent predictors of ELsys were HR (*p* = 0.00733), E (*p* = 0.159), and LVFS (*p* = 0.148), with the following regression equation:

$$ \boldsymbol{ELsys}=-41.858+0.597\times HR+0.155\times E+45.328\times LVFS $$

(adjusted *R* ^2^ 0.2087, *p* = 0.1594) (Table 4).

### Mean diastolic energy loss

ELdia, the mean value of diastolic phase EL, is a useful parameter for assessing cardiac condition that is pathological during diastolic phase, such as with mitral stenosis or aortic regurgitation. The range of ELdia was from 7.9 to 86 mW/m (mean 30.41 ± 16.93 mW/m; male 29.93 ± 17.57, female 32.96 ± 13.75; Table 2). No significant difference was observed between male and female mean values. The reference range of ELdia was 8.31 ~ 80.36 mW/m. ELdia showed a statistically significant correlation with E (*r* = 0.542, *p* < 0.0001) (Table 3 and Fig. 3). According to the multivariate analysis, the independent predictors of ELdia were E (*p* < 0.0001), HR (*p* = 0.00037), and age (*p* = 0.147), with the following regression equation:

$$ \boldsymbol{ELdia}=-39.773+0.583\times E+0.753\times HR-0.582\times age $$

(adjusted *R* ^2^ 0.4365, *p* = 0.1479) (Table 4).

### Mean kinetic energy over one cardiac cycle

KEcycle, the mean value of KE over one cardiac cycle, is a useful parameter for assessing the ejection of blood flow from the left ventricle into the left ventricular outflow tract. The range of KEcycle was from 200 to 851.6 mW/m (mean 449.74 ± 177.51 mW/m; male 432.81 ± 165.85, female 538.60 ± 220.71; Table 2). No significant difference was observed between male and female mean values. The reference range of KEcycle was 203.16 ~ 833.15 mW/m. KEcycle showed a statistically significant correlation with E (*r* = 0.442, *p* = 0.001) (Table 3 and Fig. 3). According to the multivariate analysis, the independent predictor of KEcycle was E (*p* = 0.0013), with the following regression equation:

$$ \boldsymbol{KEcycle}=155.307+4.495\times E $$

(adjusted *R* ^2^ 0.1783, *p* = 0.0013) (Table 4).

### Energetic performance index

EPI, the ratio of KEcycle to ELcycle, is a useful parameter for assessing the energetic efficiency of the heart, especially in evaluating the success of cardiac surgery. The range of EPI was from 5.3 to 37.6 (mean 18.48 ± 7.74; male 18.98 ± 8.23, female 15.86 ± 3.66; Table 2). No significant difference was observed between male and female mean values. The reference range of EPI was 5.80 ~ 36.67.

### Reproducibility

**Fig. 4:**
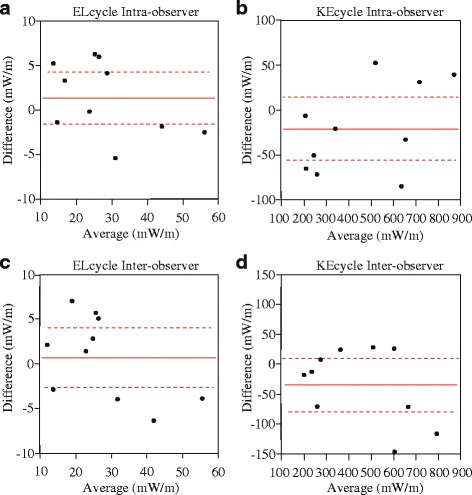
Bland–Altman plots of intra- and inter-observer variability. **a** Intra-observer variability in the average energy loss over one cycle (ELcycle). **b** Intra-observer variability in kinetic energy over one cycle (KEcycle). **c** Inter-observer variability for ELcycle. **d** inter-observer variability for KEcycle. The mean values of pairs of measurements are plotted against the difference between the measurements. The *red* continuous line represents the arithmetic mean and the *red* dotted lines represent 95% limits of agreement

Intra-observer variability for ELcycle was 15.6% ± 11.5% and the inter-observer variability was 17.0% ± 9.0%. Intra-observer variability for KEcycle was 14.4% ± 12.0% and the inter-observer variability was 13.2% ± 9.7%. The intraclass correlation coefficients (ICCs) of ELcycle and KEcycle were 0.953 and 0.978, respectively, for intra-observer measurements (95% confidence interval, 0.833–0.988 and 0.920–0.994, respectively). For inter-observer measurements, the ICCs of ELcycle and KEcycle were 0.943 and 0.947, respectively, (95% confidence interval, 0.791–0.985 and 0.791–0.987, respectively). Bland-Altman plots for each variability are shown in Fig. 4.

## Discussion

By using VFM, we were able to confirm in this study the normal pattern vortex in the left ventricle, and to define reference values for each type of EL, KE, and EPI in healthy volunteers. In future, left ventricular function and load can be assessed with regard to energetics in the perioperative period by referring to the values obtained in this study. In addition, if the reference values of the energetic parameters were established with transesophageal echocardiography, energetic values can be calculated in a few minutes by online real-time VFM and the result of surgery can be evaluated with regard to energy even during the operative period.

It is natural in the human left ventricle that there are a strong clockwise vortex under the anterior mitral leaflet and a weak counterclockwise vortex under the posterior mitral leaflet during early diastole. In our analysis, although the weak counterclockwise vortex quickly diminished, the strong clockwise vortex continued until the isovolumic contraction phase, as reported previously [2, 5, 20–23]. The vortex enables efficient blood streaming and minimizes the flow dissipative EL [24–27]. However, heart valvular disease, ischemic heart disease, dyssynchrony of the left ventricle, or mitral valve replacement surgery can alter the intraventricular vortex configuration and increase EL [8, 9, 20, 22, 28–31]. Increased EL stresses the heart and impairs cardiac function [9, 10]. Although it is useful to calculate EL in order to assess the cardiac load, EL decreases in cases of severe deterioration of cardiac function [8]. Conversely, even in cases of normal heart function, EL increases in the hyperdynamic state. Therefore, it is important to take account of KE in the LVOT in conjunction with EL. For this reason, we defined the EPI to assess how efficiently the left ventricle ejects blood into the LVOT.

The correlations of ELsys with HR and LVFS are reasonable because the main factor in EL generation is flow acceleration during systole, as shown in Figs. 1 and 2. The VFM images in Fig. 1 show that the velocity vectors are well aligned towards the outflow during the systolic phase, and the EL images in Fig. 2 demonstrate that high EL (the bright area) is concentrated in the outflow during this phase. The source of flow acceleration is left ventricular contractility enhanced by an increment in HR [32]. Obviously, LVFS is the index of left ventricular contractility. It is also reasonable that E is an independent predictor of ELsys because the preload provides the left ventricular contractility according to the Frank–Starling law.

A factor of EL generation during diastole is flow dissipation, as shown in Figs. 1 and 2. The VFM images in Fig. 1 show that the velocity vectors form vortex in the left ventricle during the diastolic phase, and the EL images in Fig. 2 demonstrate that the area of high EL (the bright area) spreads over the left ventricle during this phase. The greater the value of E, the more dissipative is the intraventricular flow. The other independent predictors of ELdia were HR and age. An increment in HR leads to a short diastolic time, which makes the flow more turbulent. Aging has an effect on diastolic function, resulting in the transmitral flow becoming more hypodynamic, resulting in a decrease in ELdia.

Because ELcycle combines both ELsys and ELdia, it is affected by the factors of both the systolic and diastolic phases.

As discussed earlier, the preload provides left ventricular contractility according to the Frank–Starling law. Thus, during the main portion of diastolic flow, E is correlated with KEcycle.

EPI is useful for assessing patients with various heart diseases and for evaluating cardiac surgery performance. Because EL and KE are susceptible to hemodynamic condition, either can sometimes be problematic for assessing cardiac condition. However, EPI enables the diagnosis of cardiac condition, such as hyperdynamic, overloaded, impaired, or decompensated. EPI is a new index, and data about EPI will need to be collected across a range of cardiac conditions.

### Perspective of the energetic parameters

In practical situations, there are various indicating parameters for operative therapy. For example, peak aortic valve velocity, mean pressure gradient, symptoms and left ventricular ejection fraction in the case of aortic stenosis, while vena contracta, regurgitant volume, symptoms, left ventricular ejection fraction, left ventricular diastolic diameter, and left ventricular systolic diameter in the case of aortic regurgitation. The most appropriate timing for operative therapy can be challenging using these parameters, particularly in mixed pathological conditions such as aortic stenosis combined with aortic regurgitation. However, assessing EL allows the estimation of the cardiac workload with ease as a single parameter. Like EL, KE plays an important role in assessing left ventricular function [33, 34]. In the present study, we focused particularly on the KE in the LVOT, which enabled us to assess left ventricular systolic function similar to cardiac output. Furthermore, we have been able to assess the results of surgery in terms of energetic efficiency using EPI during perioperative period, not only with TTE, but also with transesophageal echocardiography during the intraoperative period.

Recently, good outcomes of early surgery have been reported, suggesting that a surgical intervention should be performed before cardiac function deteriorates [35–38]. Periodical follow-up and energetic parameter calculations with echocardiography can indicate the optimum period when KE or EPI starts to decline.

### Limitations

The participants of the study were potentially different from the general population because they volunteered to participate in the study and had differing characteristics from those who did not volunteer. Indeed, the mean participants’ age was approximately 30 years, and 84% of the participants were male. The values of energetic parameters may have been affected by both age-related decrease in cardiac function and gender differences.

The sample size of the present study was small for reference ranges that were calculated using 2.5% and 97.5% values. Although the present study is the first to report reference ranges for each energetic parameter using VFM, further studies are required.

The regression equations of this study showed lower adjusted R^2^ values than those in the study of children by Hayashi et al. [18]. This is because each energetic parameter is difficult to predict for the following reasons. First, HR of adults is independent of age and lower than that of children, with less effect on each energetic parameter. Second, because the left ventricular area of adults is larger than that of children, flow velocity can change easily and the proportion of laminar flow and turbulent flow is variable. It is important to define the reference range for each energetic parameter rather than simply to present the regression equation, because adults can be classified into a single category (different from children) and what matters most is to assess the patient’s cardiac condition quickly during the perioperative period, particularly intraoperatively.

### An example of the intraoperative application of energetic parameters

**Fig. 5:**
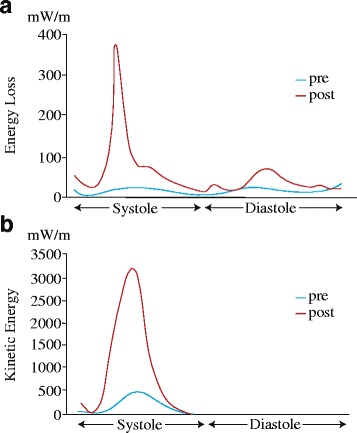
Trans-catheter aortic valve replacement performance. Graphs of energy loss (**a**) and kinetic energy (**b**) over one cardiac cycle before and after trans-catheter aortic valve replacement due to severe aortic valve stenosis. Both energy loss and kinetic energy were low before the procedure because the left ventricular inflow and outflow were stagnated due to the stenotic aortic valve. After the procedure, the left ventricular inflow and outflow became dynamic, and both energy loss and kinetic energy increased. The increase in kinetic energy exceeded the increase in energy loss resulting in an increase in the energy performance index, indicating improvement of the cardiac function

As an example of intraoperative energetic parameters calculation, we describe the case of an 80-year-old woman who underwent trans-catheter aortic valve replacement due to severe aortic valve stenosis. The procedure was performed under general anesthesia from a right femoral approach with the successful implantation of a 26-mm Sapien XT (Edwards Lifesciences, Irvine, CA). We performed TTE in the operation room before and after the procedure. The transaortic valve flow maximum velocity was 4.8 m/s and the left ventricular wall motion before the procedure was diffuse hypokinesis. After the procedure, the transprosthetic valve flow maximum velocity was 2.3 m/s, and the left ventricular wall motion was normal. Before the procedure, ELcycle, KEcycle, and EPI were 9.5 mW/m, 63.9 mW/m, and 5.76, respectively. After the procedure, these changed to 40.7 mW/m, 458.3 mW/m, and 11.26, respectively. ELcycle increased after the procedure because the flow stagnation due to the stenotic aortic valve was released. However, KEcycle increased more than ELcycle, thus improving the energetic efficiency (EPI) (Fig. 5).

## Conclusion

EL, KE, and EPI reference values were defined using VFM. These reference values enable the assessment of a range of cardiac conditions in any clinical situation. EPI is useful for assessing patients with various heart diseases and for evaluating the success of cardiac surgery. However, further investigation of EPI is required to validate its efficacy.

## Abbreviations

A: Peak blood flow velocity at the mitral valve during late diastole
a’: Peak myocardial velocity at the septal mitral annulus during late diastole
BSA: Body surface area
E: Peak blood flow velocity at the mitral valve during early diastole
e’: Peak myocardial velocity at the septal mitral annulus during early diastole
EL: Energy loss
ELcycle: Mean energy loss over one cardiac cycle
ELdia: Mean diastolic energy loss
ELsys: Mean systolic energy loss
EPI: Energetic performance index
HR: Heart rate
KE: Kinetic energy
KEcycle: Mean kinetic energy over one cardiac cycle
LVFS: Left ventricular fractional shortening
LVOT: Left ventricular outflow tract
TTE: Transthoracic echocardiography
VFM: Vector flow mapping

## References

[1] Kilner PJ, Yang GZ, Wilkes AJ, Mohiaddin RH, Firmin DN, Yacoub MH (2000). Asymmetric redirection of flow through the heart. Nature.

[2] Rodriguez Munoz D, Moya Mur JL, Fernandez-Golfin C, Becker Filho DC, Gonzalez Gomez A, Fernandez Santos S (2015). Left ventricular vortices as observed by vector flow mapping: main determinants and their relation to left ventricular filling. Echocardiogr.

[3] Kilner PJ, Henein MY, Gibson DG (1997). Our tortuous heart in dynamic mode--an echocardiographic study of mitral flow and movement in exercising subjects. Heart Vessels.

[4] Sengupta PP, Khandheria BK, Korinek J, Jahangir A, Yoshifuku S, Milosevic I (2007). Left ventricular isovolumic flow sequence during sinus and paced rhythms: new insights from use of high-resolution Doppler and ultrasonic digital particle imaging velocimetry. J Am Coll Cardiol.

[5] Hong GR, Pedrizzetti G, Tonti G, Li P, Wei Z, Kim JK (2008). Characterization and quantification of vortex flow in the human left ventricle by contrast echocardiography using vector particle image velocimetry. JACC Cardiovasc Imaging.

[6] Charonko JJ, Kumar R, Stewart K, Little WC, Vlachos PP (2013). Vortices formed on the mitral valve tips aid normal left ventricular filling. Ann Biomed Eng.

[7] Martinez-Legazpi P, Bermejo J, Benito Y, Yotti R, Perez Del Villar C, Gonzalez-Mansilla A (2014). Contribution of the diastolic vortex ring to left ventricular filling. J Am Coll Cardiol.

[8] Agati L, Cimino S, Tonti G, Cicogna F, Petronilli V, De Luca L (2014). Quantitative analysis of intraventricular blood flow dynamics by echocardiographic particle image velocimetry in patients with acute myocardial infarction at different stages of left ventricular dysfunction. Eur Heart J Cardiovasc Imaging.

[9] Stugaard M, Koriyama H, Katsuki K, Masuda K, Asanuma T, Takeda Y (2015). Energy loss in the left ventricle obtained by vector flow mapping as a new quantitative measure of severity of aortic regurgitation: a combined experimental and clinical study. Eur Heart J Cardiovasc Imaging.

[10] Bahlmann E, Gerdts E, Cramariuc D, Gohlke-Baerwolf C, Nienaber CA, Wachtell K (2013). Prognostic value of energy loss index in asymptomatic aortic stenosis. Circulation.

[11] Pedrizzetti G, La Canna G, Alfieri O, Tonti G (2014). The vortex--an early predictor of cardiovascular outcome?. Nat Rev Cardiol.

[12] Honda T, Itatani K, Takanashi M, Mineo E, Kitagawa A, Ando H (2014). Quantitative evaluation of hemodynamics in the Fontan circulation: a cross-sectional study measuring energy loss in vivo. Pediatr Cardiol.

[13] Sengupta PP, Pedrizzetti G, Kilner PJ, Kheradvar A, Ebbers T, Tonti G (2012). Emerging trends in CV flow visualization. JACC Cardiovasc Imaging.

[14] Rodriguez Munoz D, Markl M, Moya Mur JL, Barker A, Fernandez-Golfin C, Lancellotti P (2013). Intracardiac flow visualization: current status and future directions. Eur Heart J Cardiovasc Imaging.

[15] Hong GR, Kim M, Pedrizzetti G, Vannan MA (2013). Current clinical application of intracardiac flow analysis using echocardiography. J Cardiovasc Ultrasound.

[16] Garcia D, Del Alamo JC, Tanne D, Yotti R, Cortina C, Bertrand E (2010). Two-dimensional intraventricular flow mapping by digital processing conventional color-Doppler echocardiography images. IEEE Trans Med Imaging.

[17] Itatani K, Okada T, Uejima T, Tanaka T, Ono M, Miyaji K (2013). Intraventricular flow velocity vector visualization based on the continuity equation and measurements of vorticity and wall shear stress. Jpn J Appl Phys.

[18] Hayashi T, Itatani K, Inuzuka R, Shimizu N, Shindo T, Hirata Y (2015). Dissipative energy loss within the left ventricle detected by vector flow mapping in children: Normal values and effects of age and heart rate. J Cardiol.

[19] Lang RM, Badano LP, Mor-Avi V, Afilalo J, Armstrong A, Ernande L (2015). Recommendations for cardiac chamber quantification by echocardiography in adults: an update from the American Society of Echocardiography and the European Association of Cardiovascular Imaging. J Am Soc Echocardiogr.

[20] Faludi R, Szulik M, D’Hooge J, Herijgers P, Rademakers F, Pedrizzetti G (2010). Left ventricular flow patterns in healthy subjects and patients with prosthetic mitral valves: an in vivo study using echocardiographic particle image velocimetry. J Thorac Cardiovasc Surg.

[21] Chen M, Jin JM, Zhang Y, Gao Y, Liu SL (2013). Assessment of left ventricular diastolic dysfunction based on the intraventricular velocity difference by vector flow mapping. J Ultrasound Med.

[22] Rodriguez Munoz D, Lozano Granero C, Luis ZJ (2015). Vector flow mapping in mitral valve disease: a novel method for the assessment of flow mechanics and their potential implications for mitral valve repair. Curr Cardiovasc Imaging Rep.

[23] Zhang H, Ren X, Song J, Cao X, Wang B, Liu Y (2016). Intraventricular Isovolumic Relaxation Flow Patterns Studied by Using Vector Flow Mapping. Echocardiogr.

[24] Bolger AF, Heiberg E, Karlsson M, Wigstrom L, Engvall J, Sigfridsson A (2007). Transit of blood flow through the human left ventricle mapped by cardiovascular magnetic resonance. J Cardiovasc Magn Reson.

[25] Zhang H, Zhang J, Zhu X, Chen L, Liu L, Duan Y (2012). The left ventricular intracavitary vortex during the isovolumic contraction period as detected by vector flow mapping. Echocardiogr.

[26] Zhang H, Liu L, Chen L, Ma N, Zhou L, Liu Y (2013). The evolution of intraventricular vortex during ejection studied by using vector flow mapping. Echocardiogr.

[27] Pedrizzetti G, Domenichini F (2005). Nature optimizes the swirling flow in the human left ventricle. Phys Rev Lett.

[28] Pedrizzetti G, Domenichini F, Tonti G (2010). On the left ventricular vortex reversal after mitral valve replacement. Ann Biomed Eng.

[29] Itatani K (2014). When the blood flow becomes bright. Eur Heart J.

[30] Rodriguez Munoz D, Moya Mur JL, Lozano Granero C, Fernandez-Golfin C, Zamorano Gomez JL (2015). Flow collision in early aortic ejection: an additional source of kinetic energy loss in patients with mitral prosthetic valves. Eur Heart J Cardiovasc Imaging.

[31] Kakizaki R, Nabeta T, Ishii S, Koitabashi T, Itatani K, Inomata T (2016). Cardiac resynchronization therapy reduces left ventricular energy loss. Int J Cardiol.

[32] Ross J, Miura T, Kambayashi M, Eising GP, Ryu KH (1995). Adrenergic control of the force-frequency relation. Circulation.

[33] Nogami Y, Ishizu T, Atsumi A, Yamamoto M, Kawamura R, Seo Y (2013). Abnormal early diastolic intraventricular flow ‘kinetic energy index’ assessed by vector flow mapping in patients with elevated filling pressure. Eur Heart J Cardiovasc Imaging.

[34] Jeong D, Anagnostopoulos PV, Roldan-Alzate A, Srinivasan S, Schiebler ML, Wieben O (2015). Ventricular kinetic energy may provide a novel noninvasive way to assess ventricular performance in patients with repaired tetralogy of Fallot. J Thorac Cardiovasc Surg.

[35] Enriquez-Sarano M, Avierinos JF, Messika-Zeitoun D, Detaint D, Capps M, Nkomo V (2005). Quantitative determinants of the outcome of asymptomatic mitral regurgitation. N Engl J Med.

[36] Kang DH, Kim JH, Rim JH, Kim MJ, Yun SC, Song JM (2009). Comparison of early surgery versus conventional treatment in asymptomatic severe mitral regurgitation. Circulation.

[37] Suri RM, Aviernos JF, Dearani JA, Mahoney DW, Michelena HI, Schaff HV (2011). Management of less-than-severe mitral regurgitation: should guidelines recommend earlier surgical intervention?. Eur J Cardiothorac Surg.

[38] Speir A, Henry LL, Hunt SL, Holmes SD, Ad N (2013). Health-related quality of life following isolated aortic valve surgery: is earlier intervention better?. J Heart Valve Dis.

