# Erratum to: Vector flow mapping analysis of left ventricular energetic performance in healthy adult volunteers

**DOI:** 10.1186/s12872-017-0612-4

**Published:** 2017-06-30

**Authors:** Koichi Akiyama, Sachiko Maeda, Tasuku Matsuyama, Atsushi Kainuma, Maki Ishii, Yoshifumi Naito, Mao Kinoshita, Saeko Hamaoka, Hideya Kato, Yasufumi Nakajima, Naotoshi Nakamura, Keiichi Itatani, Teiji Sawa

**Affiliations:** 1 Department of Anesthesiology, Kyoto Prefectural University of Medicine, 465 Kajii-cho, 602-8566, Kamigyo, Kyoto, Japan; 2 Emergency Medicine, Kamigyo, Japan; 3 Cardiovascular Surgery, Kyoto Prefectural University of Medicine, Kamigyo, Kyoto, Japan; 4 Department of Anesthesiology, Kansai Medical University, Hirakata, Japan; 5 Department of Statistical Genetics, Kyoto University, Kamigyo, Kyoto, Japan

## Erratum

Following publication of this article [1], it has come to our attention that the corresponding author email address was captured incorrectly as kanslik@koto.kpu-m.ac.jp. The correct corresponding author email address is kanaslik@koto.kpu-m.ac.jp.

## References

[1] Akiyama A, Maeda S, Matsuyama T, Kainuma A, Ishii M, Naito Y, Kinoshita M, Hamaoka S, Kato H, Nakajima Y, Nakamura N, Itatani K, Sawa T (2017). Vector flow mapping analysis of left ventricular energetic performance in healthy adult volunteers. BMC Cardiovasc Disord.

